# Dietary bioactive compounds targeting insulin resistance: mechanisms and preventive potential

**DOI:** 10.3389/fnut.2026.1918360

**Published:** 2026-07-29

**Authors:** Vincenzo Aiello, Mauro Lombardo, Gilda Aiello, Gianluca Tripodi, Chiara Castelli, Giulia Matarese, Manuela Tamburro, Giancarlo Ripabelli, Sara Baldelli, Serena Castelli

**Affiliations:** 1 Department for the Promotion of Human Science, Quality of Life San Raffaele Open University, Rome, Italy; 2 Department of Medicine and Health Sciences “Vincenzo Tiberio”, University of Molise, Campobasso, Italy; 3 IRCCS San Raffaele Roma, Rome, Italy

**Keywords:** low glycemic index diet, Mediterranean diet, nutraceuticals, obesity, plant-based diet, type 2 diabetes mellitus

## Abstract

Insulin resistance (IR) is a condition in which the biochemical response to insulin is impaired due to both genetic and environmental factors. Insulin is a peptide hormone released by pancreatic *β*-cells that plays a systemic role in the regulation of glucose homeostasis. Under insulin-resistant conditions, higher concentrations of insulin are required to achieve the same downstream effects, leading to hyperinsulinemia and impaired glucose tolerance. IR is associated with several metabolic abnormalities, including obesity, dyslipidemia, impaired glucose tolerance, and type 2 diabetes, and represents a key early event in the development of this disease. Moreover, IR has a very high global prevalence, which underlies the breadth of its clinical implications and the associated health impact. Targeting insulin resistance represents an important strategy not only for therapy but also for prevention. In this context, nutritional strategies and functional foods rich in bioactive compounds have emerged as promising approaches to improve insulin sensitivity. Dietary patterns characterized by a high intake of whole grains, fruits, vegetables, legumes, and unsaturated fatty acids have been associated with improved metabolic control and a reduced risk of insulin resistance. Moreover, increasing evidence suggests that specific dietary bioactive compounds, such as polyphenols, flavonoids, dietary fibers, omega-3 fatty acids, and other plant-derived metabolites, may exert beneficial metabolic effects by modulating key mechanisms involved in IR, including oxidative stress, chronic low-grade inflammation, gut microbiota composition, and cellular signaling pathways regulating glucose and lipid metabolism. In this review, we evaluate emerging nutritional strategies and bioactive compounds involved in the prevention and management of IR, which consequently have significant implications at the public health level in terms of risk prevention and improved prognosis.

## 1 Introduction

Insulin is a peptide hormone synthesized and secreted by pancreatic *β*-cells within the islets of Langerhans. It plays a central role in maintaining glucose homeostasis by promoting glucose uptake from the bloodstream into key metabolic tissues such as the liver, adipose tissue, and skeletal muscle. Beyond its effects on glucose metabolism, insulin orchestrates a broad range of anabolic and regulatory processes, including glycogen synthesis, lipid metabolism, DNA replication, gene transcription, amino acid uptake, as well as protein synthesis and turnover (1). Consequently, its role in systemic homeostasis and metabolic regulation is pivotal. Insulin secretion is initiated when glucose levels exceed approximately 5 mM, whereas more moderate fluctuations in the 2–4 mM range are sufficient to stimulate its biosynthesis. Following food intake, increased glucose metabolism promotes a coordinated endocrine response, characterized by enhanced insulin production from pancreatic *β*-cells and suppression of glucagon release from *α*-cells, ultimately restoring circulating glucose levels to physiological range (2). The process by which glucose intake leads to insulin synthesis is referred to as glucose-induced insulin secretion. Under normal physiological conditions, elevations in plasma glucose levels trigger a proportional increase in insulin secretion, resulting in higher circulating insulin concentrations. This, in turn, facilitates glucose uptake into peripheral tissues while concurrently suppressing hepatic gluconeogenesis and glucose output, thereby contributing to the restoration of systemic glucose homeostasis. Insulin resistance (IR) is defined as a condition in which insulin-stimulated glucose uptake in skeletal muscle and adipose tissue is impaired, together with a reduced ability of insulin to suppress hepatic glucose production. Clinically, IR is associated with a broad spectrum of disorders, including obesity, type 2 diabetes mellitus (T2DM), metabolic syndrome, cardiovascular diseases, metabolic dysfunction–associated fatty liver disease (MAFLD), polycystic ovary syndrome (PCOS), and cancer (1, 3). Indeed, IR is characterized by impaired insulin-mediated regulation of blood glucose, reduced glucose utilization, dysregulated lipid metabolism, increased ectopic lipid accumulation, and enhanced lipolytic activity in adipocytes. It is often accompanied by compensatory hyperinsulinemia aimed at maintaining glycemic control. Given the highly systemic role of insulin and its impact on multiple organs, disruption of this glucose homeostasis mechanism leads to widespread and multifaceted physiological consequences. For these reasons, IR represents a central pathogenic mechanism underlying numerous metabolic disorders. More broadly, any disease or condition that disrupts normal metabolic processes can be classified as a metabolic disease, posing a significant threat to human health and substantially impacting quality of life.

The pathogenesis of IR arises from a complex interplay between genetic predisposition and environmental influences. Its development is driven by multiple interconnected mechanisms, including alterations in the internal milieu, such as chronic inflammation, hypoxia, lipotoxicity, and immune dysregulation, as well as broader metabolic disturbances affecting key tissues and circulating metabolites (3). Genetic determinants of IR encompass defects at multiple levels of insulin biology and metabolic regulation. These include structural abnormalities of insulin itself, alterations in insulin receptor function and downstream signaling pathways, and mutations affecting key regulators of glucose and lipid metabolism. For instance, rare variants of insulin exhibit reduced bioactivity and impaired receptor binding. Similarly, mutations in the insulin receptor gene can lead to severe hereditary forms of IR, as observed in syndromes such as type A IR, Donohue syndrome, and Rabson–Mendenhall syndrome. Beyond these rare monogenic conditions, IR more commonly arises from the combined effect of multiple genetic variants. These involve genes regulating glucose transport and sensing (e.g., GLUT4, glucokinase), nuclear receptors controlling metabolic homeostasis (e.g., PPAR family), and components of lipid metabolism, including adipokines such as leptin, adiponectin, and resistin (3, 4). In addition to genetic determinants, environmental factors play a crucial role in the onset and progression of IR. These include a broad spectrum of lifestyle-related components, with diet representing a major contributor, particularly in terms of caloric excess, nutrient composition, and eating patterns. Physical activity is another key modulator, as regular exercise enhances insulin sensitivity, whereas sedentary behavior promotes metabolic dysfunction. Aging further contributes to the decline in metabolic efficiency, often exacerbating IR (5). Together, these factors interact dynamically with genetic predisposition, shaping individual susceptibility to metabolic impairment. Moreover, these environmental factors not only modulate insulin sensitivity but can also act as direct triggers for the onset of IR itself.

Despite their causal and contributory role in IR, environmental factors also represent key points of intervention, both for its prevention and for therapeutic management. In this context, this review will examine the mechanistic basis and preventive potential of dietary bioactive compounds in modulating IR, with a particular focus on their ability to target key metabolic, inflammatory, and signaling pathways involved in its onset and progression.

## 2 Clinical relevance and public health burden of IR

IR is among the major threats to health in the modern world. The main factors contributing to its widespread prevalence include the increased consumption of calorie-dense, low-fiber fast foods and the decline in physical activity. A recent systematic review reported a global IR prevalence of 26.53%, with values ranging between 26 and 30% across different populations and geographical areas (6). Indeed, IR is highly prevalent in regions such as the United States (U. S.), where unhealthy dietary habits are more common. A recent analysis of the National Health and Nutrition Examination Survey (NHANES) highlighted that approximately 40% of U. S. adults aged 18–44 years exhibit IR, confirming the public health relevance of this condition, which is not limited to obese individuals (7). Furthermore, a rising trend in both hyperinsulinemia and IR was reported among adolescents. Data from a nationally representative cohort of U. S. adolescents without diabetes or pre-diabetes derived from NHANES (1999–2020) demonstrated a significant increasing trend in both hyperinsulinemia and IR. In particular, prevalence of hyperinsulinemia increased from 15.2% (95%CI: 12.1–18.9) in 1999–2000 to 21.5% (95%CI: 17.1–26.3) in 2017–2020. Similarly, IR defined by HOMA-IR rose from 14.0% (95%CI: 11.1–17.8) to 20.4% (95%CI: 16.4–25.6), corresponding to an increase of 3.4% per NHANES two-year cycle (*p* < 0.05). Although both obese and overweight adolescents showed a higher prevalence of hyperinsulinemia and an elevated HOMA-IR compared with normal-weight subjects, a significant increase over time was observed only in the overweight cohort (8). Overall, these findings may reflect the contribution of increased urbanization and lifestyle changes among adolescents, particularly in settings where exposure to obesogenic environments and sedentary behaviors is more pronounced (9). Therefore, metabolic dysregulation appears early in life, as reported by a cross-sectional study including young people aged 14–18 years in Slovakia, which identified a baseline IR prevalence of 18.6%, especially among patients with high body weight. Beyond anthropometric associations, this study showed links between metabolic impairment and detrimental behavioral phenotypes that included physical inactivity, heavy consumption of sugar-sweetened beverages, omitting breakfast, and irregular eating patterns, reinforcing the impact of modern nutritional habits on European adolescents (10). Further evidences indicate that pediatric and adolescent population represents a vulnerable group, characterized by an early risk of developing metabolic abnormalities and progressing toward chronic diseases in the early stages of life (11). Conversely, data from northern Ghana revealed an IR prevalence of 7.6% among adult population, with a marked gender disparity (9.9% women versus 4.5% men). These epidemiological patterns illustrate that global IR prevalence ranges from low levels (7–10%) in traditional rural African environments to high rates (25–45%) in urbanized and Western societies, depending on diagnostic metrics and underlying metabolic environment (12), and areas characterized by a higher degree of urbanization are associated with higher levels of HOMA-IR (13). However, recent data underline that metabolic disorders and IR display a remarkably steep acceleration even in low- and middle-income countries that are undergoing rapid epidemiological shifts and urban sprawl (14).

IR and the resulting hyperglycemia represent risk factors for the development of metabolic and chronic degenerative diseases, including T2DM (15). However, the global prevalence of IR is estimated to be three times higher than that of diabetes worldwide (16). According to the 11th edition of the IDF Diabetes Atlas, in 2024, approximately 11.1% of the global adult population -over 500 million individuals- were living with diabetes, with projections to 900 million cases by 2050. In addition, the World Health Organization estimates that diabetes will become the seventh leading cause of death worldwide by 2030 (17), and will be associated to high economic burden. In a study with projections from 2015 to 2030 (18) was reported that diabetes-related costs are expected to rise dramatically in the next decades in terms of both direct healthcare expenditures and indirect costs associated with productivity loss, disability, and premature mortality, to which contributes the growing IR prevalence. Consequently, preventive strategies targeting the early stages of insulin dysregulation play a crucial role, not only for a clinical perspective, but also for ensuring the long-term sustainability of healthcare systems. In this context, updating the evidence on lifestyle interventions for diabetes prevention is urgently needed, especially in countries where limited healthcare resources make the evaluation of real-world intervention effectiveness particularly important. However, the implementation of lifestyle interventions in real-world settings was rarely addressed (19). Consequently, there is an urgent need to develop and implement lifestyle interventions which are effective, feasible and sustainable, and actively engaging all relevant stakeholders (20). Beyond T2DM, IR is associated with a broader spectrum of conditions, including obesity, cardiovascular diseases, non-alcoholic fatty liver disease (NAFLD), metabolic syndrome, and polyendocrine metabolic ovarian syndrome (PMOS). Therefore, the economic impact includes direct management of these conditions and their long-term complications.

Since IR is a condition primarily driven by inadequate nutrition, functional foods and nutraceuticals offer a promising dietary approach to addressing nutrition-related diseases. Bioactive compounds in foods are under investigation for elucidating their antihyperglycemic effects and benefits in alleviating chronic diseases and protecting human health. These compounds act on chronic inflammation, oxidative stress, gut microbiota composition, and glucose metabolism, thus are studied in the context of prevention strategies for both IR and T2DM (21). From a clinical perspective, IR is associated with worsening metabolic status and increased likelihood of complications. In individuals with severe obesity, IR appears to correlate more strongly with lobular inflammation than with other histopathological components of NAFLD, suggesting a potential relationship between hepatic inflammation and impaired systemic insulin signaling (22). Emerging evidences support a strong bidirectional relationship between IR and major depressive disorder (MDD); thus, IR could have importance in the field of metabolic psychiatry (23). In addition, T2DM exerts not only peripheral but also central effects, contributing to neuropsychiatric symptoms and cognitive decline, likely accelerating the progression of Alzheimer’s disease and other forms of dementia. These effects are associated with brain IR, which may also occur independently of T2DM itself (24).

Hence, dietary strategies represent one of the most effective interventions for improving insulin sensitivity and lowering progression toward severe metabolic disorders. An effective nutritional strategy may include the consumption of flavanols, bioactive compounds with beneficial effects on IR and cognitive function. In particular, regular flavanol consumption is associated with a reduction in certain markers of age-related cognitive decline, likely through improvements in insulin sensitivity (25).

IR as a risk factor for chronic diseases is well documented, particularly for cardiovascular diseases. It was demonstrated that higher HOMA-IR values are associated with progressively greater cardiovascular risk, with a significant dose–response relationship (26). This burden will further increase with growing T2DM prevalence if no preventive actions are undertaken (27). A recent study showed that IR represents a determinant associated with an increased risk of multiple comorbidities and mortality, with stronger associations in females. The analysis with a median follow-up of 13 years on a cohort of 429,159 participants from the UK Biobank, identified robust associations between IR and 26 diseases, including glycometabolic disorders, atherogenic dyslipidemia, gout, and Parkinson’s disease-related neurodegeneration. Progressive IR increases were associated with 31, 18, and 8% higher risk of pancreatitis, sleep disorders, and bacterial infections, respectively (28). Therefore, IR should not be considered as a pre-pathological condition, being a modifiable health determinant and targeted interventions have impact on the distribution of related diseases.

Dietary patterns based on functional nutrients represent one of the principal population-level preventive strategies. The integration of bioactive compounds and functional foods, particularly flavonoids, represents a versatile and powerful preventive tool. The regular use of these molecules in the diet acts as a primary prevention strategy, which can shift the collective risk curve by improving insulin sensitivity and modulating low-grade inflammation before disease onset. The effectiveness of this approach is supported by findings from the EPIC-InterAct study (29), in which high flavonoid intake significantly reduced T2DM incidence (30). It was also demonstrated that polyphenols exert beneficial effects on several aspects of energy metabolism and body weight regulation, reducing body mass index (BMI) (31). Furthermore, the use of nutraceutical compounds fully fits within the framework of quaternary prevention, encompassing actions for protecting individuals from excessive or unnecessary medical interventions, while promoting acceptable alternatives. In the overmedicalization era, flavonoids use could be considered a safe strategy for managing individuals with early metabolic alterations, avoiding pharmacological therapies. This approach further protects patients from the potential adverse effects of polypharmacy, and simultaneously supports the economic sustainability of healthcare systems. Therefore, promoting functional nutritional models rich in polyphenols is a public health priority aimed at reducing healthcare costs related to chronic complications, and ensuring greater long-term metabolic resilience.

## 3 Molecular and cellular mechanisms of IR

IR is a multifactorial condition in which insulin-sensitive tissues progressively lose their ability to respond appropriately to insulin, leading to alterations in both glucose and lipid metabolism. At its core lies an integrated impairment of insulin signaling, tightly linked to lipotoxicity, oxidative stress, and mitochondrial dysfunction. These interconnected processes collectively contribute to the progressive metabolic deterioration characteristic of type 2 diabetes.

### 3.1 Insulin signaling

Under physiological conditions, the liver maintains blood glucose levels during fasting through glycogenolysis and gluconeogenesis (32). Following food intake, pancreatic *β*-cells secrete insulin, which promotes glucose uptake and storage in peripheral tissues, thereby favoring anabolic processes over catabolic ones (33, 34).

At the cellular level, insulin binds to its receptor, a receptor tyrosine kinase (IRTK), inducing autophosphorylation of tyrosine residues and the recruitment of adaptor proteins such as insulin receptor substrates (IRS1 and IRS2) (35). Phosphorylated IRS proteins activate phosphatidylinositol 3-kinase (PI3K), leading to the production of phosphatidylinositol-3,4,5-trisphosphate (PIP3). PIP3 recruits Akt to the plasma membrane, where it is activated by phosphoinositide-dependent kinase 1 (PDK1) and the mechanistic target of rapamycin complex 2 (mTORC2) (36), thereby initiating the phosphorylation of multiple downstream substrates in skeletal muscle, liver, and adipose tissue, and mediating the metabolic effects of insulin.

In skeletal muscle, insulin signaling through Akt promotes GLUT4 translocation to the plasma membrane and glycogen synthesis (37). Akt activates members of the Rab GTPase family, which regulate vesicular trafficking, as well as Ras-related C3 botulinum toxin substrate 1 (RAC1), which is involved in actin cytoskeleton remodeling (38).

In the liver, insulin-activated Akt2 phosphorylates forkhead box O1 (FOXO1), resulting in suppression of gluconeogenesis (39). Concurrently, insulin promotes glycogen synthase activity, thereby enhancing glycogen synthesis (40).

In adipose tissue, insulin suppresses lipolysis, reducing the availability of gluconeogenic substrates (41).

### 3.2 IR

IR is a pathological condition in which the responsiveness of insulin-sensitive tissues to the hormone is reduced, leading to a decreased ability of peripheral tissues to respond to physiological insulin levels and a compensatory increase in insulin secretion to maintain metabolic homeostasis. In this condition, the major effects of insulin, including glucose uptake, inhibition of gluconeogenesis, and suppression of lipolysis, are impaired (35). This reflects a dysregulation of insulin signaling caused by alterations in multiple molecular pathways.

Defects in the PI3K/Akt pathway reduce glucose uptake and increase hepatic glucose production and lipolysis, thereby contributing to hyperglycemia and metabolic dysfunction (42–44). In parallel, impaired AMPK signaling promotes lipid accumulation, inflammation, and oxidative stress (45). Increased activation of JNK-1 further contributes to IR by promoting serine phosphorylation of IRS-1, thereby inhibiting PI3K/Akt signaling (46). This results in reduced glucose uptake and altered lipid metabolism. In addition, JNK activation promotes inflammation via NF-κB, establishing a self-amplifying loop between stress, inflammation, and IR (47, 48).

In insulin-resistant states, pancreatic *β*-cells initially compensate by increasing insulin secretion in response to higher peripheral demand; however, prolonged stimulation leads to oxidative stress and progressive functional exhaustion (47, 49). Free fatty acids (FFAs), acting through receptors such as GPR40, modulate insulin secretion under physiological conditions, but in the context of lipotoxicity they contribute to *β*-cell dysfunction (50). Moreover, islet amyloid polypeptide (IAPP) deposition induces β-cell loss, a process further exacerbated by activation of the RAGE receptor, which promotes oxidative stress, inflammation, and apoptosis (49, 51, 52).

In skeletal muscle, the primary site of insulin-stimulated glucose disposal, IR is mainly associated with impaired GLUT4 translocation to the plasma membrane, resulting in reduced glucose uptake (53). Notably, alternative glucose transport pathways, such as those mediated by AMPK during exercise or hypoxia, remain largely preserved (54), indicating that the primary defect lies within the insulin signaling cascade. Accordingly, early defects are observed at the level of the insulin receptor (IR), IRS1, PI3K, and AKT, including reduced receptor tyrosine kinase activity and decreased IRS1 phosphorylation and PI3K activation (55).

In the liver, IR impairs the regulation of glucose metabolism, leading to increased gluconeogenesis and contributing to fasting hyperglycemia (56, 57). This is characterized by failure to suppress the transcription factor FOXO1, together with increased availability of gluconeogenic substrates derived from inadequately suppressed adipose tissue lipolysis (58). In parallel, hepatic glycogen synthesis is reduced both in fasting and postprandial states in patients with T2DM (56).

In adipose tissue, IR is mainly characterized by loss of control over lipolysis, resulting in increased release of free fatty acids that fuel hepatic gluconeogenesis and contribute to systemic metabolic dysfunction (58). This establishes a self-reinforcing loop in which excess lipid substrates further worsen insulin sensitivity.

A further relevant aspect is selective IR, in which specific signaling branches remain active or become overactivated despite overall impaired insulin action. In the liver, this manifests as an inability of insulin to suppress glucose production while preserving its capacity to stimulate lipogenesis, thereby contributing to hyperglycemia, hyperlipidemia, and hepatic steatosis (59).

### 3.3 Lipotoxicity, oxidative stress, and mitochondrial dysfunction

IR is driven by a bidirectional interplay between alterations in lipid metabolism and chronic low-grade inflammation (60). In this context, lipotoxicity represents a central mechanism in the pathogenesis of IR and arises from caloric excess, lipid accumulation, and increased lipolysis, ultimately leading to impaired cellular homeostasis in peripheral tissues (61, 62). The concept of “pan-lipotoxicity” further extends this process to a systemic dysregulation of lipid metabolism (63).

A large body of evidence indicates that ectopic lipid accumulation in peripheral tissues, particularly liver and skeletal muscle, is a key determinant of IR, even in the absence of visceral obesity (64). Consistently, a high proportion of obese patients with T2DM present non-alcoholic fatty liver disease (NAFLD), which is strongly associated with hepatic IR and diabetes (65). Animal models further support the causal role of ectopic lipid accumulation, as high-fat diets or lipid infusions induce IR in liver and muscle (66). A clinically relevant example is lipodystrophy, in which impaired adipose tissue storage capacity leads to lipid redistribution toward liver and muscle, resulting in severe IR (67).

The primary mechanism through which ectopic lipids impair insulin signaling involves bioactive lipid intermediates, particularly diacylglycerols (DAGs) and ceramides. Lipid spillover promotes the accumulation of these intermediates, especially in skeletal muscle, contributing significantly to reduced glucose uptake (68, 69). In particular, ceramides inhibit the Akt/PKB pathway and promote cellular stress, inflammation, and apoptosis, and are strongly associated with insulin-resistant states (70, 71). According to the DAG–PKC model, DAG accumulation activates protein kinase C (PKC), particularly novel PKC (nPKC) isoforms, which interfere with insulin signal transduction. In the liver, DAG activates PKCε, which translocates to the membrane and inhibits insulin receptor tyrosine kinase activity, thereby reducing IRS-2/PI3K/Akt2 signaling (72).

Excess lipid accumulation is closely linked to chronic low-grade inflammation (“metaflammation”), mediated by adipokines and pro-inflammatory cytokines (IL-1, IL-6, TNF-*α*) (36). Toll-like receptor 4 (TLR4), activated by saturated fatty acids, promotes the activation of inflammatory kinases (JNK, IKK, p38), leading to serine phosphorylation of IRS1 and impaired insulin signaling (73). In addition, TLR4 links inflammation and lipotoxicity by stimulating ceramide biosynthesis (74). JNK, PKC, and IKK pathways not only directly interfere with insulin receptor signaling but also activate NF-κB, thereby amplifying the inflammatory response (75, 76). NF-κB is further enhanced under hyperglycemic conditions via receptor for advanced glycation end-products (RAGE) activation, which increases the expression of pro-inflammatory cytokines (TNF-*α*, IL-1β, IL-6) and amplifies endoplasmic reticulum stress and reactive oxygen species (ROS) production (77, 78). Collectively, these processes converge to inhibit insulin signaling through serine phosphorylation of insulin receptor substrates and reduced GLUT4 expression (79–81).

Saturated fatty acids, particularly palmitic acid (PA), contribute to the development of IR through a central role of oxidative stress, which acts as an integrating hub between impaired insulin signaling, metabolic dysfunction, and inflammatory activation. In particular, PA increases the production of reactive oxygen species (ROS) through activation of NADPH oxidase and upregulation of NOX2 and NOX4 isoforms (82). This increased oxidative burden directly impairs the IRS/PI3K/Akt/eNOS signaling pathway, reducing nitric oxide bioavailability and compromising insulin-dependent cellular functions (83).

There is a strong link between mitochondrial dysfunction and oxidative stress. Several studies associate ectopic lipid accumulation and IR with reduced mitochondrial capacity for lipid oxidation (84). Insulin-resistant individuals exhibit reduced ATP synthesis in skeletal muscle, increased intramyocellular lipid accumulation, decreased mitochondrial density, and impaired oxidative phosphorylation, even in young and lean individuals predisposed to type 2 diabetes (85).

Mouse models with IRS-1 and IRS-2 alterations display both IR and mitochondrial dysfunction, including reduced oxidative phosphorylation and abnormal mitochondrial morphology, mediated by the IRS/PI3K/FOXO pathway (86).

In addition, lipotoxic conditions induce excessive mitochondrial fission mediated by DRP1, which impairs insulin-stimulated glucose utilization (87). Alterations in mitochondrial dynamics, particularly a shift toward fission, are associated with increased ROS production, reduced ATP synthesis, impaired *β*-oxidation, and decreased insulin-dependent glucose uptake (88). Finally, impaired mitophagy, a selective form of autophagy responsible for mitochondrial turnover, is closely linked to IR and T2DM, as it regulates the removal of damaged mitochondria via the PINK1–Parkin pathway, activated by loss of membrane potential and increased ROS levels (89, 90).

Overall, lipotoxicity, inflammation, and oxidative stress are tightly interconnected processes that converge on impaired insulin signaling. Therefore, effective therapeutic strategies should simultaneously target multiple pathways, including inflammation, oxidative stress, mitochondrial function, and TLR4-mediated signaling, in order to improve insulin sensitivity (36).

## 4 Epigenetic and signaling modulation of insulin by nutrients

**Figure 1:**
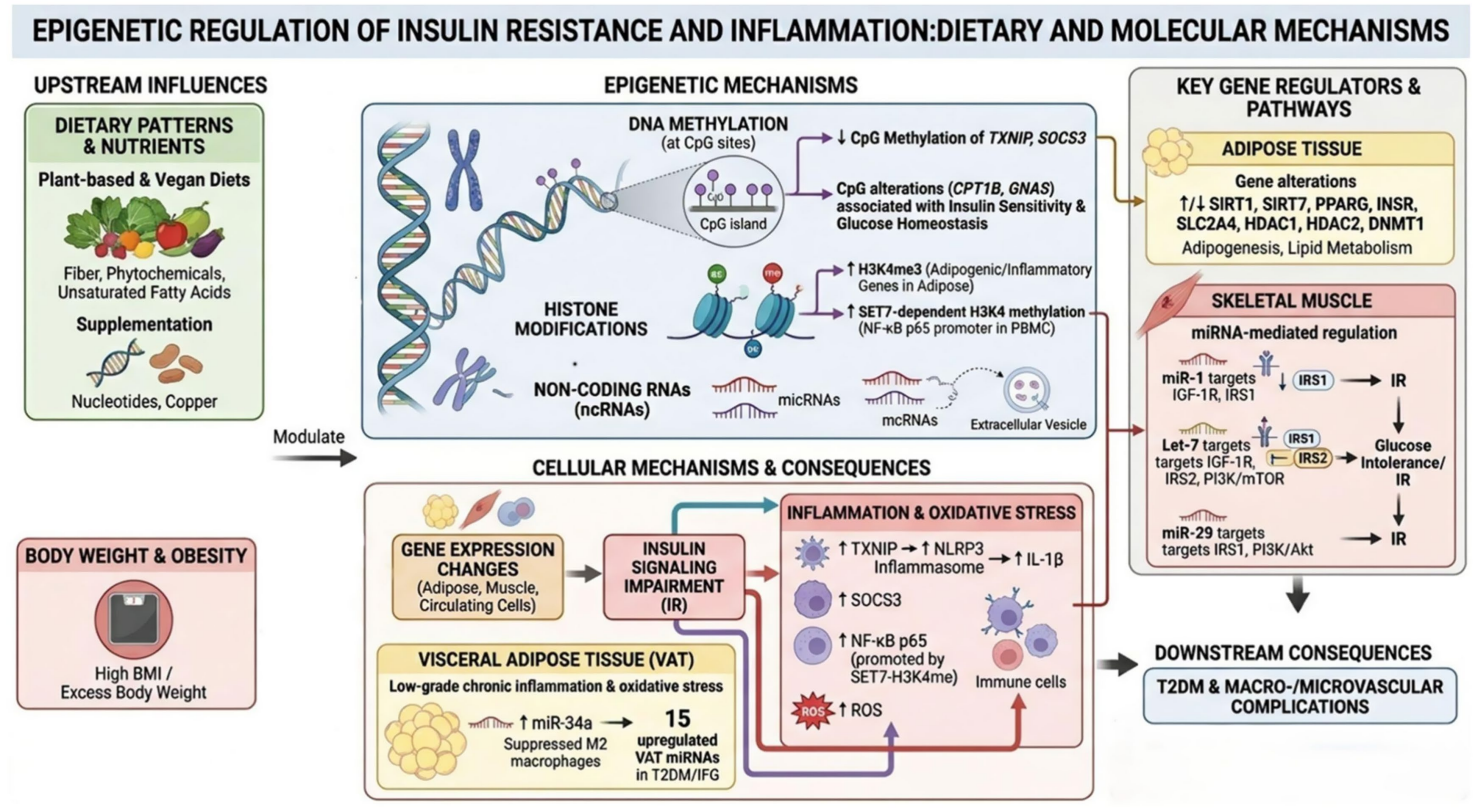
Epigenetic mechanisms regulating IR and glucose metabolism through DNA methylation, histone modifications, and microRNA signaling. The illustration depicts how dietary and environmental factors drive epigenetic alterations at key metabolic loci, thereby exacerbating IR. Gene-specific histone modifications (H3K4me3) and altered expression of metabolic regulators (SIRT1, PPARG, INSR, SLC2A4) further contribute to adipogenic dysfunction and impaired insulin signaling. Concurrently, dysregulated microRNAs modulate macrophage polarization and directly inhibit insulin signaling pathway components (IGF-1R, IRS proteins, PI3K/Akt), establishing a multi-layered epigenetic basis for IR progression and T2DM risk.

Recent evidence has demonstrated that dietary patterns can profoundly influence gene expression and the epigenetic regulation of insulin signaling and glucose metabolism. In this context, multiple examples have been reported: specific CpG sites, including those within CPT1B and GNAS, have been associated with insulin sensitivity and glucose homeostasis (91, 92). Moreover, nutritional factors such as nucleotide supplementation and trace element intake, particularly copper, have been shown to modulate DNA methylation at CpG loci linked to the risk of T2DM (93). Epigenetic marks, including DNA methylation, histone modifications, and regulation by noncoding RNAs (ncRNAs), modulate gene expression without altering the underlying DNA sequence (Figure 1). Increasing evidence indicates that these mechanisms contribute to the pathogenesis of numerous diseases, not only metabolic but also neoplastic (94). Regarding T2DM and its macro- and microvascular complications, epigenetic modifications are now emerging as promising therapeutic targets in this disease (95). Epigenetic alterations have also been associated with systemic and cellular consequences of IR, such as inflammation and oxidative stress. A reduced methylation level of the TXNIP gene, a key regulator of oxidative stress, has been associated with the progression of IR and T2DM, as well as with enhanced inflammatory responses. Upregulation of TXNIP promotes activation of the NLRP3 inflammasome, leading to increased release of pro-inflammatory cytokines, such as IL-1β. Moreover, recent evidence indicates that hypomethylation-induced overexpression of SOCS3 further contributes to IR development and increases the risk of T2DM (96). Excess body weight, and consequently a high BMI and IR, induce changes in DNA methylation that are associated with increased production of the pro-inflammatory factor NF-κB in circulating immune cells. Obesity, in fact, promotes systemic inflammation, even in the presence of normal fasting glycemia, and this inflammatory state may precede the onset of IR itself (97). In individuals with T2DM, peripheral blood mononuclear cells exhibit SET7 methyltransferase–dependent H3K4 methylation at the NF-κB p65 promoter, representing a key epigenetic modification associated with a pro-inflammatory phenotype. Notably, inhibition of SET7 activity attenuates NF-κB–driven inflammatory and pro-oxidative signaling (98).

Consistent with these observations, increased body weight–associated epigenetic alterations have been linked to histone modifications: specifically, elevated H3K4me3 levels in the promoter regions of adipogenic and inflammatory genes in human adipose tissue are positively correlated with BMI and HOMA-IR indices, further supporting the connection between epigenetic regulation, inflammation, and IR (99). Moreover, epigenetic mechanisms can directly influence insulin signaling pathways, thereby modulating IR. In adipose tissue, no global differences in histone modifications have been observed between IR and insulin-sensitive individuals. However, gene-specific alterations have been reported, particularly affecting key regulators of insulin signaling such as SIRT1 and SIRT7. These changes are also associated with differential expression of genes involved in insulin sensitivity, adipogenesis, and lipid metabolism, as well as epigenetic regulators including PPARG, INSR, SLC2A4, HDAC1, HDAC2, and DNMT1, demonstrating that changes in histone modifications represent an essential component of IR in adipose tissue. Plant-based and vegan dietary patterns have been associated with reduced epigenetic aging and improved insulin sensitivity, as demonstrated by DNA methylation clock analyses. These observations extend previous epidemiological evidence by providing a plausible mechanistic framework, suggesting that diets enriched in dietary fiber, phytochemicals, and unsaturated fatty acids may influence DNA methylation at specific loci involved in insulin signaling and metabolic regulation (92).

A growing body of recent research has highlighted the involvement of ncRNAs in the regulation of IR, not only at the cellular level but also as signaling molecules within extracellular vesicles. For instance, miR-34 has been shown to suppress macrophage polarization toward the anti-inflammatory M2 phenotype. In overweight individuals, increased expression of miR-34a in visceral adipose tissue (VAT) positively correlates with the incidence of IR and systemic inflammation (100). Strycharz and colleagues analyzed multiple miRNAs in normoglycemic (NG), impaired fasting glucose (IFG), and T2DM subjects, showing that several were differentially expressed. In particular, 15 out of 75 tested miRNAs were found to be upregulated in the VAT of female patients with T2DM and/or IFG compared to NG individuals (101). These alterations in miRNA expression may be associated with low-grade chronic inflammation and increased oxidative stress in the visceral adipose tissue of hyperglycemic individuals. Other miRNAs have also been implicated in the regulation of insulin signaling in skeletal muscle. For instance, miR-1, a muscle-enriched miRNA, modulates insulin signal transduction by targeting IGF-1R and IRS1; its downregulation in high-fat diet–induced obese mice is associated with impaired insulin signaling and hyperglycemia, suggesting a role as an early marker of IR. Similarly, the Let-7 family regulates insulin sensitivity by targeting IGF-1R and IRS2 and modulating PI3K/mTOR pathways; its overexpression leads to glucose intolerance and peripheral IR, whereas its inhibition improves metabolic outcomes. In addition, miR-29 negatively regulates insulin signaling by targeting IRS1 and components of the PI3K/Akt pathway, and its upregulation in insulin-resistant skeletal muscle further contributes to impaired insulin responsiveness (102).

The evidence supporting the role of epigenetics in the development of IR and related diseases also represents an important opportunity for intervention, considering that epigenetic modifications and ncRNA expression can be modulated through nutrition and nutraceutical approaches.

## 5 Oxidative stress and inflammation in IR

In recent decades, oxidative stress has emerged as a central pathogenic mechanism in the development of IR and its metabolic complications (103). It is classically defined as a homeostatic imbalance between the excessive production of reactive oxygen species (ROS) and the effectiveness of endogenous antioxidant defense systems. Under healthy conditions, low concentrations of ROS play a fundamental role in redox signaling by influencing transcriptional control (104) and cell cycle regulation (105). However, at high concentrations they become cytotoxic agents, making the boundary between physiology and pathology extremely fragile (106). A crucial aspect highlighted by recent literature concerns the cross-talk between mitochondrial ROS and extra-mitochondrial redox enzymes, in particular NADPH oxidases (NOXs). In contexts of hyperglycemia or excess of free fatty acids (FFA), a vicious circle is triggered in which mitochondrial ROS activate NOXs, which in turn further increase ROS production. In this scenario, H_2_O_2_ acts as a real redox switch, capable of amplifying oxidative stimulation (107).

In particular, to understand the impact of oxidative stress on IR, it is essential to analyze the insulin receptor signaling cascade. Glucose transporter 4 (GLUT4) is the main mediator of glucose uptake in insulin-sensitive tissues (108). Prolonged exposure to high insulin concentrations induces a reduction of GLUT4 on the cell surface. This mechanism stimulates the pancreas to produce more insulin, which in turn exacerbates peripheral tissue desensitization and chronic down-regulation of the transporter (109). At the molecular level, the enzyme NADPH oxidase 4 (NOX4) plays a very important role. When insulin levels exceed the optimal range, a functional switch occurs in phosphoinositide 3-kinase (PI3K): instead of acting on the canonical substrate phosphatidylinositol 4,5 bisphosphate (PIP2), the enzyme begins to phosphorylate the Rac protein, drastically enhancing the oxidant activity of NOX4 (110). The excess of ROS thus produced determines the activation of casein kinase-2 (CK2), the stimulation of the retromer protein complex, which instead of promoting the recycling of GLUT4 toward the membrane, diverts its traffic toward the trans-Golgi network and, finally, toward lysosomes for proteolytic degradation. This molecular diversion ensures that glucose levels remain high despite the abundance of insulin, fueling a pro-oxidant environment. In this signaling pathway, mitochondria also play a fundamental role, contributing to an increase in the ROS load due to nutrient-rich environments. Indeed, under such conditions, the electron transport chain becomes saturated in the attempt to produce ATP. This state of mitochondrial hyperactivity causes a massive production of ROS (111), which damage cellular macromolecules and activate specific mediators of stress and inflammation. Among the latter we find nuclear factor kappa-light-chain-enhancer of activated B cells (NF-kB) c-Jun N-terminal kinases (JNK) (112) and p38 MAPK (113) which directly interfere with insulin signaling.

As previously mentioned, IR is closely linked to a state of chronic inflammation characterized by an imbalance between pro-inflammatory mediators (such as tumor necrosis factor-alpha (TNF-*α*) and the interleukins IL-6 and IL-1*β*) and anti-inflammatory molecules such as IL-10 and TGF-1 (114). In the context of IR, adipose tissue and recruited immune cells produce an excess of cytokines that act in a paracrine manner on adipocytes and hepatocytes. This inflammatory stimulus activates a series of kinases, including IκB kinase β (IKKβ), JNK, S6 kinase (S6K), and mTOR (115, 116). These Enzymes induce inhibitory phosphorylation of serine residues on the IRS-1 protein, blocking the downstream signaling cascade (PI3K/Akt pathway). The molecular result is a drastic reduction in glucose uptake and glycogen synthesis in the liver and muscles (116). This creates a vicious cycle in which inflammation promotes IR, and IR, by promoting adiposity, further fuels cytokine production (117, 118). Furthermore, the impact of cytokines on insulin sensitivity is mediated by interconnected intracellular pathways, particularly the NF-kB pathway and Toll-like receptor (TLR) signaling. This in turn induces the transcription of inflammatory genes such as TNF-*α* and IL-6 that impair insulin receptor function (119, 120). IKKβ activates NF-κB by phosphorylating and degrading its inhibitor, IκB, allowing NF-κB to translocate to the nucleus and initiate the expression of pro-inflammatory genes (121). This pathway promotes cytokine production in adipocytes, hepatocytes, and macrophages, particularly in the context of obesity or a high-fat diet (120). In parallel, Toll-like receptor signaling increases NF-κB activation and cytokine overproduction, particularly through Toll-like receptor 4 and the adaptor protein MyD88.

In summary, IR is the product of a complex interplay between inflammatory signals, oxidative stress, and bioactive lipid species. In this scenario, lipids stimulate inflammation, and cytokines, in turn, alter lipid metabolism, perpetuating metabolic damage. Understanding these interconnected networks is essential for developing therapeutic strategies aimed at restoring metabolic balance and counteracting the complications of IR.

## 6 Dietary patterns associated with improved insulin sensitivity

### 6.1 Mediterranean dietary pattern and insulin sensitivity

The Mediterranean diet (MD) is among the most extensively studied dietary patterns in relation to insulin sensitivity. Evidence from randomized controlled trials (RCTs) suggests that its metabolic effects are mediated by multiple mechanisms, including improvements in dietary fat quality, reductions in low-grade inflammation, modulation of hepatic lipid metabolism, and alterations in gut microbiota composition.

Several RCTs indicate that the MD may improve insulin sensitivity independently of weight loss. In individuals with non-alcoholic fatty liver disease (NAFLD), a randomized crossover trial demonstrated a significant increase in insulin sensitivity measured by hyperinsulinaemic-euglycaemic clamp, accompanied by a reduction in hepatic steatosis, without differences in body weight compared with a low-fat/high-carbohydrate diet (122). Similarly, an isocaloric MD intervention in overweight individuals induced changes in the gut microbiome and circulating metabolome, including metabolites related to fibre fermentation, which were associated with metabolic improvements potentially linked to insulin sensitivity (123).

The benefits of the MD appear particularly relevant in individuals at high cardiometabolic risk. In the PREDIMED-Plus trial, an intensive lifestyle intervention based on an energy-restricted MD combined with physical activity significantly improved IR and glycaemic control in older adults with overweight/obesity and metabolic syndrome (124). In a shorter-term RCT, adherence to an MD also reduced fasting insulin and improved body composition in young overweight adults, suggesting metabolic benefits beyond clinical populations (125).

However, findings are not entirely consistent. Some trials report improvements in lipid profiles without significant changes in insulin sensitivity. For instance, a controlled intervention comparing Mediterranean and high-monounsaturated-fat diets in adults with mild abdominal obesity found no effect on insulin sensitivity despite improvements in serum lipids (126). Likewise, in a randomized crossover trial, a low-fat vegan diet improved body weight, lipid concentrations, and insulin sensitivity more than a Mediterranean diet, whereas the Mediterranean diet had a greater effect on blood pressure (127).

Emerging evidence indicates that the metabolic response to the MD may also depend on genetic background. In the CORDIOPREV study, improvements in glucose homeostasis and insulin sensitivity were modulated by variants in the NLRP3 inflammasome, highlighting a potential nutrigenetic interaction (128). In addition, modifications of the MD enriched in polyphenols have been associated with systemic benefits. In the DIRECT PLUS trial, a polyphenol-rich “Green-MED” diet was associated with reduced age-related brain atrophy, with improvements in insulin sensitivity among the factors most strongly linked to this effect (129).

Further evidence in NAFLD suggests that a calorie-restricted MD improves hepatic fat accumulation and metabolic parameters, with potential additional benefits when combined with antioxidant supplementation, possibly through modulation of oxidative stress pathways (130).

Overall, randomized evidence supports a beneficial effect of the MD on insulin sensitivity, particularly in individuals with IR, metabolic syndrome, or NAFLD. The magnitude of the effect appears to depend on clinical context, intervention duration, energy balance, and comparator diet. The evidence derived from randomized controlled trials investigating the effects of the Mediterranean diet on insulin sensitivity is summarized in Table 1.

**Table 1:** Randomized controlled trials evaluating the effects of the Mediterranean diet on insulin sensitivity and related metabolic outcomes.

Authors (Year)	Study type	Dietary	Main mechanism	Main outcomes	References
Meslier et al. (123)	RCT, parallel (8 weeks)	Isocaloric Mediterranean diet vs. habitual diet	Gut microbiota modulation; increased fibre fermentation; metabolome changes (urolithins, bile acids)	↓ plasma cholesterol; microbiome remodeling; insulin sensitivity improvements associated with microbial taxa	(123)
Ryan et al. (122)	RCT, crossover (6 weeks)	Mediterranean diet vs. low-fat/high-carbohydrate diet	Hepatic lipid reduction; improved insulin signaling	↑ insulin sensitivity (clamp); ↓ hepatic steatosis independent of weight loss	(122)
Salas-Salvadó et al. (124)	RCT (12 months)	Energy-restricted Mediterranean diet + physical activity vs. control	Weight loss–dependent and independent effects; inflammation reduction; adipokine modulation	↓ IR; ↓ HbA1c; improved cardiometabolic risk factors	(124)
Barnard et al. (127)	RCT, crossover (16 weeks)	Mediterranean diet vs. low-fat vegan diet	Differences in energy density, fat quality, and fibre intake	Vegan diet > Mediterranean diet for insulin sensitivity; Mediterranean diet > vegan diet for blood pressure.	(127)
Kaplan et al. (129)	RCT (18 months)	Mediterranean diet vs. Green-MED vs. healthy dietary guidelines	High polyphenol intake; reduced oxidative stress; metabolic–neuro axis	Improved insulin sensitivity associated with reduced brain atrophy	(129)
Roncero-Ramos et al. (128)	RCT (3 years)	Mediterranean diet vs. low-fat diet	Nutrigenetic interaction (NLRP3 inflammasome); inflammation modulation	↑ insulin sensitivity only in specific NLRP3 genotypes (non-diabetic subjects)	(128)
Kabthymer et al. (125)	RCT (8 weeks)	Mediterranean diet vs. AGHE	Body composition improvement; metabolic regulation	↓ fasting insulin; ↓ body fat; ↑ lean mass	(125)
Bos et al. (126)	RCT, parallel (8 weeks)	Mediterranean diet vs. MUFA vs. SFA diet	Fat quality (MUFA vs. SFA); lipid metabolism	No significant change in insulin sensitivity; improved lipid profile	(126)
Abenavoli et al. (130)	RCT (6 months)	Mediterranean diet ± antioxidant supplementation	Oxidative stress reduction; hepatic lipid metabolism modulation	↑ insulin sensitivity (greater with antioxidants); ↓ hepatic steatosis	(130)

Summary of randomized controlled trials assessing the impact of the Mediterranean diet on insulin sensitivity and related metabolic outcomes. Variability may be explained by differences in intervention characteristics, population features, and methodological approaches. AGHE; Australian Guide to Healthy Eating.

### 6.2 Plant-based dietary patterns and insulin sensitivity

IR is a central feature of type 2 diabetes and cardiometabolic disease, and dietary composition plays a key role in its modulation. RCTs provide substantial evidence that plant-based dietary patterns, particularly whole-food and low-fat variants, improve insulin sensitivity and glycaemic control.

Several trials demonstrate improvements in IR markers following plant-based interventions. Low-fat vegan diets have been shown to reduce fasting insulin and HOMA-IR, alongside reductions in body weight, fat mass, and visceral adiposity (131–133). These effects are partly mediated by changes in body composition, although associations independent of energy intake—such as dietary acid load and macronutrient composition—have also been reported (133).

Further evidence comes from clinical populations. In individuals with type 2 diabetes, a randomized controlled trial showed that a whole-food plant-based lifestyle intervention significantly improved HbA1c, fasting insulin, and HOMA-IR, while reducing medication use and inducing partial remission in a subset of participants (134).

Mechanistic studies support these findings. Trials using hyperinsulinaemic-euglycaemic clamps have demonstrated improvements in insulin sensitivity following dietary interventions involving plant protein patterns (135, 136). In addition, plant-based interventions have been associated with reductions in fasting insulin across diverse clinical settings, including cancer and neurological disease populations (137, 138).

Acute randomized crossover studies provide insight into underlying physiological mechanisms. Plant-based meals generally elicit greater postprandial incretin responses, particularly glucagon-like peptide-1 (GLP-1), along with increased insulin secretion and improved beta-cell function compared with macronutrient-matched animal-based meals (139, 140). These effects may contribute to improved glycaemic regulation and satiety. Additional evidence indicates that plant-based meals influence neuroendocrine pathways involved in appetite regulation and insulin dynamics (141).

Alterations in amino acid composition may represent an additional mechanism. Diets higher in plant protein, typically characterized by lower branched-chain amino acid content, have been associated with improvements in IR and metabolic profiles (131). Short-term controlled-feeding studies also report trends toward improved HOMA-IR and distinct metabolomic adaptations following increased plant protein intake, although effects may be modest (142).

Not all trials demonstrate the superiority of plant-based diets. Some studies report comparable metabolic improvements between plant and animal protein diets or only modest changes in insulin sensitivity (135, 142). These findings suggest that total dietary quality, energy balance, and weight change may play a dominant role, and that not all plant-based dietary patterns exert uniform metabolic effects.

Overall, randomized evidence supports a beneficial role of plant-based diets in improving insulin sensitivity, particularly when implemented as whole-food, low-fat dietary patterns. The observed effects are likely mediated by a combination of reduced adiposity, increased fibre intake, altered amino acid composition, and enhanced incretin response, although heterogeneity across studies remains substantial. The randomized controlled trials evaluating plant-based dietary patterns and insulin sensitivity are summarized in Table 2.

**Table 2:** Randomized controlled trials evaluating the effects of plant-based dietary patterns on insulin sensitivity and related metabolic outcomes.

Study type	Dietary	Main mechanism	Main outcomes	References
Parallel RCT (16 wk)	Low-fat vegan	↓ adiposity, ↓ visceral fat	↓ HOMA-IR, ↑ insulin sensitivity	(132)
Parallel RCT (16 wk)	Plant-based (protein-focused)	↓ BCAA intake	↓ HOMA-IR, ↓ fat mass	(131)
Parallel RCT (16 wk)	Vegan (acid load)	↓ dietary acid load	↓ HOMA-IR, ↑ insulin sensitivity	(133)
Parallel RCT (24 wk)	Whole-food plant-based lifestyle	multi-factor (diet + exercise)	↓ HbA1c, ↓ insulin, ↓ HOMA-IR	(134)
Parallel RCT (6 wk)	High plant vs. animal protein	↓ methionine/BCAA	↑ insulin sensitivity (both diets)	(136)
Parallel RCT (6 wk)	High plant vs. animal protein	amino acid composition	no major difference in insulin sensitivity	(135)
Parallel RCT (8 wk)	Whole-food plant-based	↓ weight, hormonal changes	↓ fasting insulin, ↓ IR	(138)
Parallel RCT (12 mo)	Low-fat plant-based	↓ weight, lipids	↓ insulin (secondary outcome)	(137)
Crossover acute RCT	Vegan vs. meat meal	↑ incretin (GLP-1)	↑ insulin secretion, ↑ beta-cell function	(140)
Crossover acute RCT	Vegan vs. processed meat	↑ gut hormones	↑ GLP-1, ↑ insulin response	(139)
Crossover acute RCT	Vegan vs. conventional meal	neuroendocrine signaling	↑ GLP-1, altered insulin dynamics	(141)
Crossover RCT (4 wk)	↑ plant protein (flexitarian)	Amino acid and metabolomic changes	Trend ↓ HOMA-IR; modest metabolic improvements	(142)

Data are derived from randomized controlled trials conducted in adults. Outcomes include direct measures of insulin sensitivity (e.g., hyperinsulinaemic-euglycaemic clamp) and surrogate indices such as fasting insulin and HOMA-IR.

### 6.3 Low-glycemic-index dietary patterns and insulin sensitivity

Low-glycaemic index (GI) diets represent a dietary strategy aimed at mitigating postprandial fluctuations in blood glucose and insulin levels, with potential knock-on effects on insulin sensitivity. Data from randomized controlled trials suggest that modulating the quality of carbohydrates, rather than just the quantity, may influence glucose metabolism through multiple mechanisms, including reduced blood glucose variability, decreased insulin demand, and improvements in insulin action at the peripheral and hepatic levels.

Early controlled evidence has shown that lowering the dietary GI improves metabolic control in individuals with type 2 diabetes. In a randomized crossover study, Järvi et al. demonstrated that a low-GI diet significantly reduced postprandial glycaemic and insulin responses and improved peripheral insulin sensitivity, as well as having favourable effects on the lipid profile and fibrinolytic activity, indicating a broad metabolic benefit beyond glycaemic control (143). More recent studies using gold-standard methodologies have further clarified these mechanisms. In obese prediabetic subjects, Solomon et al. reported that a low-GI diet combined with physical exercise reduced postprandial hyperinsulinaemia and modulated incretin responses, particularly those of glucose-dependent insulinotropic polypeptide, suggesting a role for gut-mediated pathways in improving insulin dynamics independently of weight loss (144). Similarly, Haus et al. demonstrated that a short-term low-GI diet combined with exercise improved insulin sensitivity as measured by clamp studies, with concomitant changes in intramuscular lipid distribution, supporting a role for skeletal muscle lipid metabolism in mediating insulin action (145).

However, the magnitude of the effect is not consistent across all studies. In a randomized crossover study comparing a low-glycaemic index diet with a standard diabetic diet, Visek et al. found no significant differences in insulin sensitivity or hepatic glucose production, despite modest improvements in body composition, suggesting that the metabolic impact of manipulating the glycaemic index may be limited in certain contexts or over shorter periods (145). Broader multicentre evidence from the DIOGenes study supports a more nuanced interpretation: Goyenechea et al. demonstrated that low-GI diets, particularly when combined with a higher protein intake, were associated with better glycaemic control and a lower insulin response during the oral glucose tolerance test, while high-GI diets were linked to a worsening of HOMA-IR, highlighting the interaction between carbohydrate quality and overall dietary composition (146).

Evidence in younger populations suggests that baseline metabolic status may modulate responsiveness. In obese children, Visuthranukul et al. observed reductions in fasting insulin and HOMA-IR following a low-GI diet, although differences between groups were attenuated after adjustment, indicating that improvements may be more pronounced in individuals with greater baseline IR (147). Similarly, Iannuzzi et al. reported that a low-calorie, low-glycaemic index diet reduced IR and improved vascular markers such as carotid intima-media thickness in obese children, suggesting integrated metabolic and cardiovascular benefits (148).

Inflammatory pathways may also contribute to the metabolic effects of low-glycaemic index diets. Although changes in insulin sensitivity indices were not always significant, Rouhani et al. demonstrated that a low-glycaemic index diet reduced inflammatory markers such as hs-CRP and IL-6 in overweight adolescents, supporting the hypothesis that improvements in insulin action may be mediated, at least in part, by a reduction in low-grade inflammation (149).

In summary, evidence from randomized trials suggests that low-glycaemic-index diets may improve insulin sensitivity, particularly through a reduction in postprandial insulin requirements, modulation of incretin responses, and effects on skeletal muscle and inflammatory pathways. However, the magnitude and consistency of these effects appear to depend on baseline metabolic status, study duration, co-interventions such as physical exercise, and overall dietary composition, including protein intake and energy balance. Table 3 shows that low–glycemic index (GI) and low–glycemic load dietary patterns exert variable but generally favourable effects on insulin sensitivity across different populations.

**Table 3:** Low–glycemic index diets and insulin sensitivity: Evidence from randomized controlled trials.

Study type	Dietary	Main mechanism	Main outcomes	References
RCT, crossover	Low-GI vs. conventional diabetic diet	Reduced postprandial glycaemia and insulin demand	↑ insulin sensitivity; ↓ fasting glucose	(143)
RCT	Low-GI + exercise vs. high-GI + exercise	Improved insulin signaling and incretin response	↓ IR; ↓ hyperinsulinemia	(144)
RCT	Low-GI + exercise vs. high-GI	Reduced intramyocellular lipid accumulation	↓ HOMA-IR; ↑ insulin sensitivity	(145)
RCT	Low-GI vs. standard diabetic diet	Improved glucose kinetics	↑ insulin sensitivity (clamp)	(220)
RCT (DIOGenes)	Low-GI/high-protein vs. high-GI diets	Reduced glycaemic load and improved satiety/metabolism	↓ HOMA-IR vs. high-GI diet	(146)
RCT	Low-GI diet in obese children	Reduced glycaemic load and insulin demand	↑ insulin sensitivity; ↓ insulin	(147)
RCT	Hypocaloric low-GI vs. standard diet	Reduced insulin demand and vascular stress	↓ HOMA-IR; improved vascular markers	(148)
RCT	Low-GI diet in adolescents	Anti-inflammatory effects	↓ hs-CRP, IL-6; no major insulin changes	(149)

Summary of randomized controlled trials evaluating the effects of low–glycemic index or glycemic load diets on insulin sensitivity and related metabolic outcomes. The table reports study design, dietary intervention, proposed mechanisms, and main findings. Overall, low-GI diets tend to improve insulin-related outcomes in insulin-resistant populations or when combined with lifestyle interventions, whereas effects are less consistent in controlled isocaloric settings or healthy populations.

## 7 Bioactive compounds targeting IR

Nutraceuticals and natural bioactive compounds have been increasingly investigated in the context of chronic and cardiometabolic disorders. In particular, selected nutraceuticals have been discussed in relation to metabolic syndrome, a multifactorial condition closely associated with insulin resistance, obesity, cardiovascular risk, and type 2 diabetes. More broadly, recent literature also highlights the translational interest of nutraceuticals and natural compounds in the management of different pathological conditions, supporting their relevance as bioactive agents with pleiotropic molecular effects (150). Before discussing individual classes of dietary bioactive compounds, a comparative overview is provided to summarize their main food sources, molecular targets, proposed mechanisms, and level of evidence. This synthesis is intended to facilitate interpretation of the following subsections and to highlight the different degrees of translational maturity across compound classes.

### 7.1 Polyphenols and flavonoids

Among dietary bioactive compounds, polyphenols and flavonoids have attracted considerable interest because they can target several molecular processes involved in the development of IR, including oxidative stress, chronic low-grade inflammation, mitochondrial dysfunction, and impaired insulin signaling. Rather than acting through a single pathway, these compounds appear to exert pleiotropic effects on tissues that are central to glucose homeostasis, particularly liver, adipose tissue, skeletal muscle, and, in some contexts, the endothelium and gut-liver axis. Nutraceuticals may be employed as standalone interventions, as adjuvant strategies in combination with conventional therapies, including in the context of cancer (151), or in combination with other nutraceuticals, as exemplified by recent evidence on nutraceutical formulations (152). Within this broad class, resveratrol, quercetin, and catechins are among the most extensively investigated compounds in relation to IR prevention and metabolic health (153–155).

Resveratrol, a stilbene polyphenol mainly found in grapes and red wine, has been widely studied for its insulin-sensitizing potential. Preclinical evidence indicates that resveratrol can improve glucose homeostasis through activation of AMPK and SIRT1, attenuation of oxidative stress, and restoration of insulin signaling through the IRS-1/PI3K/Akt axis. In a rodent study, Kang et al. reported that resveratrol normalized insulin-stimulated Akt phosphorylation in liver and white adipose tissue under diet-induced obesity, lowered fasting glucose and insulin, and improved hepatic glycogen content, suggesting tissue-specific restoration of insulin responsiveness under insulin-resistant conditions (156). In human tissues ex vivo, Tran et al. showed that resveratrol reduced pro-inflammatory cytokine production and restored insulin signaling defects and glucose uptake in placenta, adipose tissue, and skeletal muscle exposed to inflammatory or microbial stimuli, supporting the idea that anti-inflammatory effects are mechanistically linked to improved insulin action (157). More broadly, the review by Szkudelski and Szkudelska emphasized that the antidiabetic action of resveratrol in animal models is consistently associated with AMPK/SIRT1 activation, antioxidant activity, and improved insulin sensitivity (Table 4) (153).

**Table 4:** Principal dietary bioactive compounds targeting insulin resistance: Sources, molecular targets, proposed mechanisms, and evidence level.

Bioactive compound/class	Main dietary sources	Main molecular targets	Proposed mechanisms in IR	Evidence level/translational note	Reference
Resveratrol	Grapes, red wine, berries, peanuts	AMPK, SIRT1, IRS-1/PI3K/Akt	↓ oxidative stress; ↓ inflammation; improved insulin signaling and mitochondrial function	Strong preclinical evidence; heterogeneous human data; bioavailability remains limiting	(153, 156–166)
Quercetin	Onions, apples, berries, capers, tea	AMPK, Akt, GLUT4, SIRT1, NF-κB	↑ glucose uptake; ↑ GLUT4 translocation; ↓ inflammatory and oxidative pathways	Robust mechanistic evidence; limited clinical consistency	(39, 154, 167–173)
Catechins/EGCG	Green tea, cocoa, tea products	AMPK, mitochondrial pathways, inflammatory mediators	↓ lipotoxicity; ↓ oxidative stress; improved insulin signaling	Consistent preclinical evidence; variable clinical effects	(155, 174–179)
Omega-3 PUFA	Fatty fish, seafood, algae, flaxseed, walnuts	GPR120/FFAR4, PPARs, NF-κB, resolvins	↓ inflammation; improved lipid metabolism; pro-resolving mediator production	Strong cardiometabolic evidence; direct IR effects remain inconsistent	(180–189)
Dietary fibers / prebiotics	Whole grains, legumes, fruits, vegetables, inulin, β-glucans	Gut microbiota, SCFAs, GPR41/43, gut barrier	↓ postprandial glycemia; ↑ SCFAs; ↓ endotoxemia and low-grade inflammation	Relatively strong translational support; effects depend on fiber type and dose	(190–196)
Plant-derived bioactive peptides	Soy, rice bran, hemp, lupin, black soybean, hydrolysates	DPP-IV, α-glucosidase, GLP-1, PI3K/Akt, GLUT4	DPP-IV/α-glucosidase inhibition; ↑ incretin signaling; ↑ glucose uptake	Mainly in vitro/animal evidence; human validation still limited	(197–210)
Carotenoids/astaxanthin	Carrots, tomatoes, leafy vegetables, algae, seafood	ROS pathways, mitochondria, PPARγ	Antioxidant and anti-inflammatory effects; ↓ lipid accumulation	Mainly observational/preclinical evidence; causality not fully established	(212–215)
Vitamin D and micronutrients	Fortified foods, vitamin D-rich foods, magnesium/chromium sources	VDR, insulin receptor activity, oxidative stress pathways	Supports insulin secretion/signaling; correction of deficiency states	Mixed evidence; most relevant in deficient individuals	(216–219)

Human evidence for resveratrol is promising but clearly heterogeneous. In patients with type 2 diabetes, Brasnyó et al. reported that resveratrol improved insulin sensitivity, reduced oxidative stress, and enhanced Akt pathway activation, providing early clinical support for its insulin-sensitizing properties (158). Similarly, in patients with NAFLD, Chen et al. found that resveratrol improved IR together with glucose and lipid metabolism, suggesting a particular relevance in metabolically compromised populations (159). Additional benefit has been reported in metabolic syndrome by Méndez-del Villar et al. and Starvaggi et al., who observed effects on insulin sensitivity and insulin secretion after supplementation (150, 160), and in patients with T2DM plus coronary heart disease by Hoseini et al. (161). However, not all trials have confirmed these effects. Yoshino et al. found no metabolic improvement in nonobese women with normal glucose tolerance after 12 weeks of supplementation (162), while Poulsen et al. reported no benefit of high-dose resveratrol on insulin sensitivity in obese men (163). Likewise, Timmers et al. showed that resveratrol as add-on therapy did not improve clamp-measured insulin sensitivity in subjects with well-controlled type 2 diabetes (164), and de Ligt et al. observed no effect on insulin sensitivity after 6 months in overweight adults, although HbA1c was modestly lower (165). A meta-analysis by Liu et al. concluded that resveratrol significantly reduced fasting glucose, insulin, HbA1c, and HOMA-IR in participants with diabetes, but not in nondiabetic subjects, suggesting that metabolic status may strongly influence responsiveness (166).

Quercetin, one of the most abundant dietary flavonols, has also been investigated as a modulator of IR through anti-inflammatory, antioxidant, and metabolic actions. Mechanistically, quercetin appears to act at multiple levels, including stimulation of glucose uptake, attenuation of inflammatory cell infiltration in adipose tissue, reduction of oxidative stress, and modulation of lipogenic pathways in liver. In skeletal muscle cells, Dhanya et al. showed that quercetin increased glucose uptake predominantly through AMPK and downstream p38 MAPK, rather than through the canonical insulin pathway, suggesting that it may bypass defective insulin signaling and still improve cellular glucose handling (167). In HepG2 cells, both Li et al. and Vidyashankar et al. reported that quercetin improved IR and hepatic lipid accumulation, at least in part through enhanced tyrosine phosphorylation within the insulin signaling cascade and suppression of lipogenic mediators such as SREBP-1c and FAS (168, 169).

Animal studies further support a beneficial role of quercetin in obesity-associated IR. Dong et al. demonstrated that dietary quercetin improved insulin sensitivity and glucose intolerance in high-fat-fed mice, while enhancing GLUT4 translocation and Akt signaling in adipose tissue and reducing macrophage and mast cell infiltration, with AMPKα1/SIRT1 identified as part of the mechanism (170). In obese Zucker rats, Rivera et al. reported that chronic quercetin administration ameliorated several components of metabolic syndrome, including IR and inflammatory status (171). Additional evidence points to a role in the gut-liver axis: in a mouse model of diet-induced NAFLD, Porras et al. found that quercetin decreased IR while attenuating dysbiosis, endotoxemia, TLR4/NF-κB activation, inflammasome signaling, and endoplasmic reticulum stress, suggesting that microbiota-mediated mechanisms may contribute to its metabolic effects (172). Central effects have also been described; Zhang et al. showed that quercetin improved hypothalamic insulin signaling in high-fructose-fed rats by downregulating AMPK/TXNIP and inhibiting NF-κB/NLRP3 inflammasome activation, thus linking hypothalamic inflammation to whole-body IR (173).

Catechins, especially epigallocatechin-3-gallate (EGCG) from green tea, have likewise been proposed as insulin-sensitizing compounds. Their mechanisms include suppression of oxidative stress, attenuation of lipotoxicity and inflammatory signaling, activation of AMPK, improvement of IRS-1/Akt signaling, and modulation of lipid metabolism. *In vivo*, Li et al. showed that EGCG attenuated free-fatty-acid-induced peripheral IR by decreasing oxidative stress, reducing PKCθ membrane translocation, activating AMPK, and improving insulin signaling (174). In obese and adipocyte models, Yan et al. found that green tea catechins ameliorated adipose IR by reducing ROS and improving glucose uptake, linking their effects directly to oxidative stress control (175). In hepatocytes, Zhang et al. reported that EGCG improved IR by alleviating inflammation, oxidative stress, and lipotoxicity through the GLUT2/PGC-1β/SREBP-1c/FAS pathway (176), while Mi et al. showed that EGCG restored insulin signaling and mitochondrial function in a Bmal1-dependent manner, connecting circadian regulation with metabolic homeostasis (177). Additional cellular data indicate that EGCG can reduce ROS-driven JNK/IRS1/AKT/GSK signaling abnormalities and improve glycogen synthesis in hepatocytes exposed to high glucose (178).

Overall, the literature indicates that resveratrol, quercetin, and catechins can modulate IR through overlapping but not identical mechanisms, most notably AMPK activation, restoration of PI3K/Akt signaling, suppression of oxidative stress and inflammatory pathways, and regulation of lipid handling in liver and adipose tissue. However, the transition from preclinical efficacy to robust clinical benefit remains incomplete. Across all three compounds, the strongest signals tend to emerge in subjects with pre-existing metabolic dysfunction, whereas effects in metabolically healthy individuals are weaker or absent. Differences in dose, formulation, treatment duration, bioavailability, baseline metabolic status, and study design likely explain much of the inconsistency in human trials. Therefore, while these polyphenols are biologically plausible and mechanistically compelling candidates for the prevention of IR, larger and better-standardized clinical studies are still needed before firm conclusions can be drawn about their preventive efficacy in human (155, 179).

### 7.2 Omega-3 fatty acids

Long-chain omega-3 polyunsaturated fatty acids (n-3 PUFAs), particularly eicosapentaenoic acid (EPA) and docosahexaenoic acid (DHA), have been extensively investigated for their role in modulation of insulin sensitivity and metabolic homeostasis. These fatty acids exert pleiotropic effects on key tissues involved in glucose metabolism, including liver, skeletal muscle, adipose tissue, and the immune system, through coordinated regulation of lipid metabolism, inflammation, and intracellular signaling pathways.

At the molecular level, omega-3 fatty acids improve insulin sensitivity primarily through activation of peroxisome proliferator-activated receptors (PPARs) and AMP-activated protein kinase (AMPK), leading to enhanced fatty acid oxidation and reduced ectopic lipid accumulation, a key driver of IR. In addition, omega-3 fatty acids have been shown to restore insulin signaling by improving phosphorylation of insulin receptor substrate-1 (IRS-1) and activation of the PI3K/Akt pathway, thereby promoting glucose uptake in insulin-sensitive tissues (180).

A central mechanism underlying the metabolic effects of omega-3 fatty acids is their potent anti-inflammatory activity. Chronic low-grade inflammation is a major contributor to IR, particularly in adipose tissue. EPA and DHA reduce the production of pro-inflammatory cytokines such as TNF-*α* and IL-6 through inhibition of NF-κB signaling, and act as precursors of specialized pro-resolving mediators (SPMs), including resolvins, protectins, and maresins, which actively promote the resolution of inflammation and restore tissue homeostasis (181). In this context, the identification of GPR120 (FFAR4) as a receptor for omega-3 fatty acids has provided mechanistic insight into their insulin-sensitizing effects. Activation of GPR120 inhibits inflammatory signaling in macrophages and improves insulin sensitivity in adipose tissue, highlighting a key link between lipid signaling and metabolic inflammation (182).

Preclinical studies consistently demonstrate that omega-3 fatty acids prevent or attenuate IR in models of diet-induced obesity. For instance, Flachs et al. showed that n-3 PUFAs enhance fatty acid oxidation and reduce lipid accumulation in liver and skeletal muscle, thereby alleviating lipotoxicity and improving insulin sensitivity (183). Similarly, González-Périz et al. demonstrated that DHA-derived lipid mediators reduce adipose tissue inflammation and improve insulin sensitivity in obese mice (184). These findings are further supported by studies showing improved mitochondrial function and oxidative capacity in skeletal muscle following omega-3 supplementation.

Despite strong mechanistic and preclinical evidence, clinical studies have produced heterogeneous results. Early meta-analyses suggested limited effects of omega-3 fatty acids on insulin sensitivity in individuals with type 2 diabetes, although consistent improvements in triglyceride levels were observed (185). Similarly, a large meta-analysis by Wu et al. found no clear association between omega-3 intake and reduced risk of type 2 diabetes, highlighting variability across populations and study designs (186).

More recent evidence confirms this variability. A systematic review and meta-analysis by Brown et al. reported that omega-3 supplementation does not significantly improve insulin sensitivity in the general population, although modest benefits may occur in metabolically compromised individuals (187).

Randomized controlled trials also suggest that the metabolic effects of omega-3 fatty acids depend strongly on baseline metabolic status. For example, Itariu et al. demonstrated that omega-3 supplementation improved insulin sensitivity and reduced adipose tissue inflammation in obese individuals, supporting a role in inflammation-driven IR (188). However, other trials in healthy or mildly insulin-resistant subjects have reported minimal or no effects, suggesting that omega-3 fatty acids may be more effective as a therapeutic rather than preventive intervention in high-risk populations.

Emerging evidence also indicates that omega-3 fatty acids interact with the gut microbiota, contributing to their metabolic effects. Dietary omega-3 intake has been associated with increased abundance of beneficial microbial taxa and enhanced production of short-chain fatty acids, which can improve insulin sensitivity and reduce systemic inflammation. Conversely, the gut microbiota may influence the metabolism and bioavailability of omega-3 fatty acids, highlighting a bidirectional relationship within the gut–metabolism axis (189).

Overall, omega-3 fatty acids represent a mechanistically well-supported class of dietary bioactive compounds with potential to modulate IR through anti-inflammatory, lipid-regulating, and insulin-sensitizing effects. However, the translation of these mechanisms into consistent clinical benefits remains incomplete. Variability in dose, duration, EPA/DHA composition, background diet, and individual metabolic phenotype likely contribute to the inconsistency of clinical findings. Future research should focus on identifying responsive subpopulations, optimizing intervention strategies, and elucidating the role of omega-3-derived lipid mediators in metabolic regulation.

### 7.3 Dietary fibers and prebiotics

Dietary fibers are broadly classified into soluble and insoluble forms, with soluble fibers—such as *β*-glucans, pectins, and inulin-type fructans—being particularly effective in improving metabolic outcomes due to their fermentability and viscosity.

One of the primary mechanisms by which dietary fibers improve insulin sensitivity is through the reduction of postprandial glycemic responses. Viscous soluble fibers delay gastric emptying and reduce glucose absorption in the small intestine, thereby attenuating postprandial glucose excursions and insulin demand. In addition, long-term intake of dietary fiber has been associated with improved glycemic control and reduced risk of type 2 diabetes, as demonstrated in epidemiological studies and clinical trials (190). The central role of dietary fibers in metabolic regulation is mediated by their interaction with the gut microbiota. Fermentable fibers are metabolized by colonic bacteria into short-chain fatty acids (SCFAs), primarily acetate, propionate, and butyrate, which exert multiple beneficial effects on host metabolism. SCFAs act as signaling molecules through G-protein-coupled receptors such as GPR41 and GPR43, influencing energy homeostasis, inflammation, and insulin sensitivity. In particular, butyrate has been shown to enhance mitochondrial function and increase energy expenditure, while propionate can regulate gluconeogenesis and satiety signaling (191). Experimental studies provide strong evidence for the insulin-sensitizing effects of dietary fibers and SCFAs. Gao et al. demonstrated that butyrate supplementation in mice improved insulin sensitivity and increased energy expenditure by promoting mitochondrial function and fatty acid oxidation (192). Similarly, De Vadder et al. showed that SCFAs regulate glucose homeostasis through a gut–brain neural circuit involving intestinal gluconeogenesis, which contributes to improved insulin sensitivity (193).

Prebiotics, defined as selectively fermented substrates that confer health benefits by modulating the gut microbiota, further enhance these metabolic effects. Inulin-type fructans and galacto-oligosaccharides have been shown to increase the abundance of beneficial bacterial taxa such as Bifidobacterium and *Akkermansia muciniphila*, which are associated with improved metabolic profiles. Notably, Everard et al. reported that increased abundance of *Akkermansia muciniphila* was associated with improved gut barrier function, reduced endotoxemia, and enhanced insulin sensitivity in obese mice (194).

Human intervention studies support the metabolic benefits of dietary fibers and prebiotics, although results vary depending on the type, dose, and duration of supplementation. For instance, supplementation with inulin-type fructans has been shown to improve insulin sensitivity and reduce inflammation markers in overweight and obese individuals (195). A systematic review and meta-analysis by Silva et al. reported that dietary fiber intake is associated with significant improvements in glycemic control and insulin sensitivity, particularly in individuals with type 2 diabetes or metabolic syndrome (196).

### 7.4 Plant-derived bioactive peptides

Plant-derived proteins represent a significant source of bioactive peptides capable of modulating glucose metabolism and improving insulin sensitivity, making them promising candidates for the prevention and management of IR. These peptides are typically released from parent proteins during gastrointestinal digestion, fermentation, or controlled enzymatic hydrolysis and can exert biological effects through several complementary mechanisms, including inhibition of enzymes involved in glucose metabolism, modulation of incretin degradation, improvement of intracellular insulin signaling, and attenuation of inflammatory and oxidative stress pathways associated with metabolic dysfunction. A growing body of experimental evidence indicates that proteins derived from soybean, rice bran, hemp seed, and lupin can generate peptide sequences capable of interacting with key metabolic targets implicated in IR. Food-derived bioactive peptides have therefore attracted increasing attention as nutraceutical compounds with potential antidiabetic properties, particularly because they may reduce blood glucose levels, improve insulin uptake, and inhibit enzymes involved in glucose homeostasis (197). Moreover, recent reviews emphasize that dietary protein hydrolysates from plant sources can generate peptides with multiple biological activities relevant to diabetes and obesity management, including modulation of metabolic signaling pathways and improvement of insulin sensitivity (198).

One of the most extensively investigated plant protein sources is soybean. Several peptides released from soybean proteins have demonstrated significant antidiabetic activity, particularly through inhibition of dipeptidyl peptidase-IV (DPP-IV), the enzyme responsible for the degradation of incretin hormones such as glucagon-like peptide-1 (GLP-1). The soybean peptide IAVPTGVA, commonly referred to as Soy1, has been shown to inhibit recombinant human DPP-IV with an IC50 of approximately 106 μM. In cell-based assays using intestinal Caco-2 cells, Soy1 maintained inhibitory activity with an IC50 of 223.2 μM, and experiments performed in human serum revealed that the peptide reduced circulating DPP-IV activity by 27.7% at a concentration of 100 μM and by 35.0% at 300 μM. By limiting incretin degradation, such peptides may prolong GLP-1 signaling and thereby enhance glucose-dependent insulin secretion (199, 200). In addition to enzymatic inhibition, some soybean peptides exert direct effects on cellular mechanisms associated with insulin sensitivity. The tripeptide FLV (Phe-Leu-Val) has been reported to attenuate inflammatory signaling in adipocytes exposed to TNF-*α*-induced IR. Treatment with FLV significantly reduced the production of pro-inflammatory mediators such as TNF-α, MCP-1, and IL-6 and suppressed the activation of inflammatory kinases including JNK and IKK, while stabilizing the inhibitory protein IκBα. Since chronic inflammation is a major driver of IR, the ability of this peptide to reduce inflammatory signaling translates into improved insulin responsiveness and increased glucose uptake in adipocyte models (201).

Another soybean peptide with potent metabolic activity is SWGEDWGEIW, which has been shown to improve glucose utilization in insulin-resistant 3 T3-L1 adipocytes. When applied at concentrations of 5, 10, and 20 μg/mL, this peptide increased glucose utilization by approximately 34.2, 74.5, and 86.7%, respectively. Mechanistic studies demonstrated that this activity is mediated through activation of the PI3K/Akt signaling pathway, leading to increased phosphorylation of Akt and enhanced expression and membrane translocation of the glucose transporter GLUT4. The metabolic effect of the peptide was abolished when cells were pretreated with the PI3K inhibitor LY294002, confirming the direct involvement of the canonical insulin signaling cascade (202). In addition to these small peptides, larger soybean-derived bioactive peptides such as aglycin have shown systemic antidiabetic effects *in vivo*. Oral administration of aglycin at a dose of 50 mg/kg/day for 4 weeks in high-fat diet and streptozotocin-induced diabetic mice significantly improved fasting blood glucose levels and glucose tolerance. Molecular analyses revealed that aglycin restored the phosphorylation of the insulin receptor (IR), insulin receptor substrate-1 (IRS-1), and Akt, while simultaneously increasing GLUT4 expression and translocation in skeletal muscle, thereby promoting glucose uptake and improving insulin sensitivity (203).

Another plant protein source that generates antidiabetic peptides is rice bran. Hydrolysates produced from defatted rice bran proteins have demonstrated significant inhibitory activity against DPP-IV. In one study, enzymatic hydrolysis using Umamizyme G yielded peptides with an IC50 of approximately 2.3 ± 0.1 mg/mL against DPP-IV. Subsequent purification and structural analysis identified two active dipeptides, Leu-Pro and Ile-Pro, among which Ile-Pro showed the strongest inhibitory potency with a Ki value of 0.11 mM and acted as a competitive inhibitor of the enzyme. Structural analysis suggests that the presence of proline at the C-terminal position facilitates interaction with the catalytic site of DPP-IV, a feature commonly observed in potent DPP-IV inhibitory peptides (204).

Hemp seed proteins have also emerged as a valuable source of bioactive peptides with potential antidiabetic activity. Recent investigations have identified multiple short peptides capable of inhibiting DPP-IV with relatively strong potency. Among these peptides, LPQNIPPL exhibited the highest inhibitory activity with an IC50 of approximately 0.08 mM, followed by YPYY (0.18 mM), YPW (0.18 mM), LPYPY (0.20 mM), WWW (0.22 mM), YPY (0.29 mM), YPF (0.42 mM), and WS (0.44 mM). These peptides were also shown to stimulate GLP-1 secretion in intestinal Caco-2 cells and enhance insulin release in INS-1 pancreatic *β*-cells, suggesting that their metabolic effects extend beyond enzyme inhibition and may involve modulation of the entero-insular axis (205). In addition to DPP-IV inhibition, hemp seed proteins can generate peptides that inhibit *α*-glucosidase, an enzyme responsible for the hydrolysis of complex carbohydrates into absorbable glucose. Two peptides identified from hemp protein hydrolysates, LR and PLMLP, have demonstrated α-glucosidase inhibitory activity, indicating their potential to reduce postprandial hyperglycemia by slowing carbohydrate digestion and glucose absorption in the small intestine (199).

Proteins derived from lupin (*Lupinus angustifolius*) also produce peptides capable of modulating metabolic pathways associated with IR. Peptides derived from *β*-conglutin proteins, including the β1, β3, and β6 isoforms, have been shown to enhance the expression of key genes involved in insulin signaling, such as IRS-1, PI3K p85, Akt, and GLUT4, in peripheral blood mononuclear cells obtained from patients with type 2 diabetes. At the same time, these peptides significantly reduced the expression of inflammatory mediators including inducible nitric oxide synthase (iNOS) and interleukin-1β (IL-1β), highlighting their dual metabolic and anti-inflammatory properties (206).

Finally, peptide hydrolysates obtained from black soybean proteins have demonstrated the capacity to improve insulin sensitivity through modulation of multiple intracellular pathways. In skeletal muscle C2C12 myotubes, these peptides increased glucose uptake and stimulated GLUT4 translocation to the plasma membrane. In HepG2 hepatocytes, they enhanced phosphorylation of Akt, glycogen synthase kinase-3β (GSK-3β), and the transcription factor Foxo1, while simultaneously suppressing markers of endoplasmic reticulum (ER) stress, a condition closely linked to IR. In diabetic db/db mice, oral administration of black soybean peptide hydrolysates for 5 weeks significantly improved glucose tolerance and reduced blood glucose levels, confirming the metabolic potential of these compounds *in vivo* (207).

Taken together, the available evidence indicates that plant-derived bioactive peptides exert antidiabetic activity through multiple complementary mechanisms, including inhibition of enzymes involved in glucose metabolism, enhancement of incretin signaling, restoration of insulin receptor signaling pathways, and suppression of chronic inflammation and cellular stress. Although most studies have been conducted *in vitro* or in animal models, the identification of specific peptide sequences with measurable biological activity supports the potential use of plant proteins as sources of nutraceutical peptides for the prevention of IR and type 2 diabetes.

A critical aspect that influences the physiological relevance of food-derived bioactive peptides is their bioavailability. After oral ingestion, peptides are subjected to extensive gastrointestinal digestion, enzymatic degradation, and limited intestinal absorption, and only a fraction can reach systemic circulation in an intact and biologically active form. Several studies indicate that peptide size, amino acid composition, and resistance to proteolytic enzymes are key determinants of their stability and absorption. In particular, short peptides containing proline residues often exhibit increased resistance to degradation and enhanced interaction with intestinal transporters such as PepT1. Moreover, food processing technologies, including enzymatic hydrolysis and fermentation, can enhance peptide release and bioaccessibility, thereby improving their metabolic potential (208, 209).

Moreover, emerging evidence suggests that bioactive peptides may also exert indirect metabolic effects through modulation of gut microbiota. Peptides reaching the colon can be further metabolized by microbial communities, generating secondary metabolites that influence host glucose metabolism and inflammatory responses. Conversely, the gut microbiota may contribute to the generation of bioactive peptide fragments from dietary proteins, thereby shaping their biological activity. This bidirectional interaction highlights the potential role of peptides in the gut–metabolism axis, although mechanistic insights remain limited and warrant further investigation (210). Despite the growing number of experimental studies, clinical evidence supporting the efficacy of bioactive peptides in improving IR remains limited. Variability in peptide composition, lack of standardized extraction and characterization methods, and differences in study design represent major challenges for translating preclinical findings into human applications. Furthermore, dose–response relationships, long-term safety, and interactions with other dietary components are still poorly defined (211).

#### 7.4.1 Carotenoids, vitamins, and micronutrients

In addition to polyphenols, fatty acids and peptides, a growing body of evidence highlights the role of carotenoids, vitamins, and essential micronutrients as modulators of insulin sensitivity and metabolic homeostasis. These compounds exert their effects primarily through antioxidant activity, regulation of gene expression, modulation of inflammation, and interaction with key metabolic signaling pathways, thereby contributing to the prevention of IR.

Carotenoids, including *β*-carotene, lycopene, lutein, and astaxanthin, are lipophilic pigments widely distributed in fruits and vegetables. Beyond their role as precursors of vitamin A, carotenoids have been shown to influence metabolic processes through their potent antioxidant and anti-inflammatory properties. Oxidative stress is a key driver of IR, as it impairs insulin signaling by promoting serine phosphorylation of insulin receptor substrate proteins and activating stress-related kinases such as JNK. Epidemiological studies have consistently reported inverse associations between circulating carotenoid levels and markers of IR and metabolic syndrome. For instance, Coyne et al. demonstrated that higher plasma carotenoid concentrations were associated with lower IR and reduced prevalence of metabolic syndrome components (212). Similarly, Sluijs et al. reported that higher dietary intake of carotenoids was associated with a lower risk of type 2 diabetes in a large prospective cohort (213).

Mechanistically, carotenoids may improve insulin sensitivity by reducing oxidative stress and inflammation, as well as by modulating transcription factors involved in metabolic regulation. In experimental models, astaxanthin has been shown to enhance insulin signaling and reduce lipid accumulation by improving mitochondrial function and decreasing reactive oxygen species production (214, 215). Additionally, carotenoids can influence adipocyte differentiation and lipid metabolism through regulation of PPARγ and other nuclear receptors. Despite these promising findings, clinical evidence remains limited. Variability in bioavailability, differences in dietary sources, and interactions with other nutrients may influence the metabolic effects of carotenoids in humans. Therefore, while carotenoids are strongly associated with improved metabolic health, further intervention studies are needed to establish causal relationships.

Several vitamins and micronutrients play essential roles in maintaining insulin sensitivity, acting as cofactors in metabolic pathways, regulators of gene expression, and modulators of oxidative stress and inflammation. Among these, vitamin D has received considerable attention due to its role in glucose metabolism and insulin action. Vitamin D receptors (VDR) are expressed in pancreatic *β*-cells and insulin-sensitive tissues, and vitamin D has been shown to enhance insulin secretion and improve insulin sensitivity through modulation of calcium homeostasis and inflammatory pathways. Observational studies have consistently reported an inverse association between serum 25-hydroxyvitamin D levels and IR (216). However, randomized controlled trials have yielded mixed results, with some studies showing modest improvements in insulin sensitivity and others reporting no significant effects, suggesting that baseline vitamin D status and supplementation dose are critical determinants of response.

Magnesium is another key micronutrient involved in glucose metabolism, acting as a cofactor for enzymes regulating insulin signaling and carbohydrate metabolism. Magnesium deficiency has been associated with impaired insulin receptor activity, increased inflammation, and oxidative stress. A meta-analysis by Larsson and Wolk demonstrated that higher magnesium intake is associated with a reduced risk of type 2 diabetes (217). Furthermore, magnesium supplementation has been shown to improve insulin sensitivity, particularly in individuals with hypomagnesemia or metabolic syndrome.

Chromium has also been investigated for its role in insulin action, as it enhances insulin receptor activity and glucose uptake. Although some studies suggest that chromium supplementation may improve glycemic control in individuals with type 2 diabetes, evidence remains inconsistent, and its clinical relevance is still debated (218).

In addition, vitamin E and vitamin C, as major antioxidant vitamins, may contribute to improved insulin sensitivity by reducing oxidative stress and preventing lipid peroxidation. However, clinical trials have produced inconsistent results, and their benefits appear to be context-dependent, often limited to individuals with elevated oxidative stress or micronutrient deficiencies (219).

Overall, vitamins and micronutrients play a supportive but significant role in the regulation of insulin sensitivity. Their effects are often synergistic and context-dependent, influenced by baseline nutritional status, metabolic condition, and interactions with other dietary components. While supplementation may be beneficial in deficient populations, current evidence does not support indiscriminate use for the prevention of IR in the general population.

## 8 Current knowledge gaps, inconsistencies, and future research directions

The available evidence should be interpreted according to the quality and certainty of the underlying study design. *In vitro* studies provide valuable mechanistic insights into the effects of bioactive compounds on insulin signaling, oxidative stress, inflammation, mitochondrial function, glucose uptake, and lipid metabolism; however, they remain reductionist and often rely on concentrations that may not be fully achievable in humans. Animal models offer a more integrated view of tissue-specific and systemic metabolic responses, including effects on liver, skeletal muscle, adipose tissue, gut microbiota, and inflammatory pathways, but interspecies differences in metabolism, dosage, bioavailability, and disease modeling limit direct extrapolation to human IR. Observational studies support associations between bioactive-rich dietary patterns and improved insulin sensitivity or reduced T2DM risk, although residual confounding, reverse causality, and dietary assessment bias should be considered. Randomized controlled trials provide the highest level of evidence, particularly for structured dietary patterns such as the Mediterranean, plant-based, and low-glycemic-index diets; however, clinical translation remains limited by heterogeneous populations, intervention duration, comparator diets, adherence, endpoints, bioavailability, and dosage standardization.

As previously mentioned, clinical studies conducted in humans show high heterogeneity in results. For example, regarding widely studied polyphenols such as resveratrol, some studies highlight significant improvements in insulin sensitivity and glycometabolic parameters in populations with obvious metabolic deficits, such as T2DM or MAFLD (158, 159). In contrast, other studies conducted in healthy, non-obese subjects or subjects with well-controlled diabetes have found no tangible metabolic benefits (162–164). These discrepancies are also confirmed at the level of synthesis and meta-analysis studies, which highlight how the baseline metabolic status of participants strongly influences the actual responsiveness to the intervention (166).

Similar inconsistencies and high variability also emerge in the literature for other widely studied compounds, such as carotenoids and some micronutrients (212, 213), whose protective effects appear strongly context-dependent and limited mainly to individuals with specific basic nutritional alterations or deficiencies. The effectiveness of overall dietary patterns, such as low glycemic index diets, also shows considerable variation based on the initial metabolic and phenotypic characteristics of the subjects (146, 147).

Major gaps in the current scientific literature include bioavailability and pharmacokinetics. In particular, there is still an incomplete understanding of the true bioavailability, metabolism, and kinetics of individual bioactive compounds in the human body. In the case of bioactive peptides, for example, gastrointestinal digestion processes, enzymatic degradation and limited intestinal permeability represent critical factors that drastically reduce the share of molecules capable of reaching the systemic circulation in an intact and biologically active form (208, 209).

Furthermore, many gaps concern the clinical validation of emerging compounds. Substances of great interest such as bioactive peptides of plant origin show robust efficacy and clear molecular mechanisms at the preclinical level (*in vitro* or *in vivo* in animal models) (202, 207), but suffer from the lack of large controlled clinical studies in humans capable of confirming their therapeutic efficacy and recommended dosages (211). Finally, the bidirectional interaction between bioactive compounds and gut microbiota composition also requires further investigation (210). Furthermore, although the nutritional approach is known to modulate gene expression and epigenetic signals involved in glucose metabolism (92), the long-term impact of diet on epigenetic marks and ncRNA regulation in humans requires more extensive and targeted field studies (95).

Therefore, in order to overcome these limitations and enhance the preventive and therapeutic potential of functional nutrition, future research should focus on a type of personalized nutrition, developing precise nutritional strategies based on nutrigenomics and epigenetic modulation, identifying specific patient subpopulations that show increased reactivity to different bioactive compounds based on their basal and phenotypic metabolic profile.

Furthermore, clinical studies could be conducted aimed at precisely defining the minimum effective dosages, the optimal duration of treatments and the standardization of methods of extraction and characterization of molecules. Finally, designing long-term randomized controlled trials (RCTs) in real-world settings, focusing not only on isolated molecules but on the synergistic action between functional foods, overall dietary patterns (such as the Mediterranean or plant-based diet), and structured lifestyle interventions, could also validate effective, easy-to-implement, and sustainable public health strategies.

Additional translational barriers include the limited availability of long-term safety data, especially for high-dose or combined nutraceutical formulations, and the lack of harmonized regulatory frameworks governing nutraceutical quality, purity, labeling, health claims, and batch-to-batch standardization. These aspects are particularly relevant because nutraceuticals may differ substantially in formulation, concentration of active compounds, bioaccessibility, and interaction with background diet or concomitant therapies.

## 9 Conclusion

In conclusion, IR represents a major global public health challenge, serving as a critical upstream driver for T2DM, cardiovascular diseases, and complex systemic metabolic dysfunction. Its pathogenesis involves a sophisticated interplay between genetic predisposition and environmental factors, where lipotoxicity, mitochondrial impairment, oxidative stress, and chronic low-grade inflammation converge to disrupt physiological insulin signaling and epigenetic regulation. In this scenario, primary and quaternary prevention strategies focused on nutritional interventions offer valuable, non-pharmacological avenues to address IR progression. Available evidence from well-designed randomized controlled trials suggests the implementation of structured dietary patterns, such as the Mediterranean, plant-based, and low-glycemic-index diets, may help improve insulin sensitivity, reduce systemic inflammation, and favorably modulate the gut microbiota, although individual responses remain highly variable. Furthermore, specific dietary bioactive compounds, including polyphenols (resveratrol, quercetin, catechins), omega-3 polyunsaturated fatty acids, fermentable fibers, and plant-derived bioactive peptides, exhibit multi-targeted pleiotropic effects capable of modulating impaired intracellular signaling cascades primarily in preclinical settings. While these mechanistic frameworks are compelling, translating these findings into robust clinical guidelines remains challenging. Current human trials often show inconsistencies regarding optimal dosages, compound bioavailability, and heterogeneous metabolic phenotypes, highlighting the critical need for further high-quality, large-scale clinical evidence to confirm these benefits. Consequently, prioritizing well-substantiated nutritional models rich in bioactive molecules holds the potential to favorably influence the collective risk curve, potentially reducing the socio-economic burden on healthcare systems and fostering long-term metabolic resilience at the population level.
